# A Review on the Hypotheses about Arterial Hypertension from the Viewpoint of Traditional Persian Medicine

**DOI:** 10.31661/gmj.v9i0.1065

**Published:** 2020-01-27

**Authors:** Davoud Hasani, Mohsen Bahrami, Hassan Ahanghar, Mohammad Kamalinejad, Soghrat Faghihzadeh, Majid Dadmehr

**Affiliations:** ^1^School of Medicine, Zanjan University of Medical Sciences, Zanjan, Iran; ^2^Research Institute for Islamic and Complementary Medicine, Iran University of Medical Sciences, Tehran, Iran; ^3^School of Traditional Medicine, Iran University of Medical Sciences, Tehran, Iran; ^4^Department of Cardiology, School of Medicine, Zanjan University of Medical Sciences, Zanjan, Iran; ^5^Department of Pharmacognosy, School of Pharmacy, Shahid Beheshti University of Medical Sciences, Tehran, Iran; ^6^Department of Biostatistics and Epidemiology, School of Medicine, Zanjan University of Medical Science, Zanjan, Iran

**Keywords:** Hypertension, Firdous al-Hekmat, Rabban Tabari, Persian Medicine

## Abstract

Hypertension is one of the most common chronic diseases in recent decades worldwide. It has been distinguished as the main risk factor of coronary artery disease, aortic dissection, heart failure, renal failure, cerebrovascular diseases, and death. In recent years, the global attention has been paid to complementary medicine to preventive, diagnostic and treatment strategies for hypertension, in previous studies, the history of hypertension from the viewpoint of traditional Persian medicine have been reviewed and several hypotheses raised. In this article, we present the concept of an Iranian physician namely *Ali ibn Sahl Rabban al-Tabari* on hypertension, which has not been considered properly until now. He defined a state called "*Hayajan al-dam*" that has clinical manifestations similar to hypertension, although these are not the same, also, we reviewed the viewpoint of other Persian medicine scholars namely Rhazes, Haly Abbas, Akhawayni and Avicenna for this topic.

## Introduction

Hypertension (HTN) is one of the important public health problem [1,2] and is known as the common risk factor for morbidity and mortality worldwide [1-4]. Elevated blood pressure is a major risk factor for cardiovascular diseases, stroke and chronic kidney diseases [2-4]. Proper antihypertensive therapy prevents its complications and decreased hospitalization and health care costs [2-5]. However, many patients are unresponsive to standard treatment [5], also, drug therapy is long term, costly and may cause side effects [2,6], therefore, a large number of patients not taking proper treatment as prescribed [7], some patients use complementary and alternative therapies for the management of HTN [2,5]. Traditional Persian medicine (TPM) with a long history is one of the ancient medicine which is widely used today [6], several studies have been reviewed the history of HTN from the viewpoint of well-known TPM scholars such as Akhawayni, Rhazes, Avicenna and Haly Abbas for providing a better understanding of main points of this disease [6-10]. However, the concept of *Ali ibn Sahl Rabban al-Tabari, *who have an undeniable contribution to the medical sciences and pioneer of those TPM practitioners about this topic has not been considered properly until now, he defined a state called “ *Hayajan al-Dam*”, which has clinical manifestations similar to HTN, although these are not exactly the same. The aim of this article is reviewing the concept of “ *Hayajan al-Dam*” and its treatment from the Tabari’s views and compare with current knowledge of HTN.

## Search Strategies

In this qualitative study, we read the book “ *Firdous al- Hikmat fi al- Tibb (*Paradise of Wisdom *)”* written by Tabari, in which we focus on finding and understanding his point of view. To better comprehend the subject, we reviewed the viewpoint of other Persian medicine scholars namely Rhazes, Haly Abbas, Akhawayni and Avicenna for this topic, finally, we searched in the databases such as PubMed, Scopus, Science Direct and Google Scholars related to the topic. Also, we introduced a brief account of Tabari’s life and his works. In the discussion and analysis of data, all members of the research team participated, and we tried to follow the principles of qualitative studies.

###  Biography of Rabban al-Tabari (838-870 A.D)

*Ali ibn Sahl Rabban al-Tabari* (838–870 AD) was a famous Persian physician in the Islamic golden age (early medieval time 9th-12th century AD), he was from Tabaristan (now in Iran) [11]. In the Persian literature, there is evidence that he performed human dissection first-hand and descried the embryology and anatomy of the brain, nerves, heart, and liver [11]. Rhazes was the student of Tabari [11]. Tabari is the author of “ *Firdous al- Hikmat fi al- Tibb*” (Paradise of Wisdom), this book was an essential medical encyclopedia for his time [12], which written in Arabic and mainly influenced by Greek, Syrian, Persian, and Indian sources [11,13] and is one of the oldest medical texts in the Islamic world [12,13]. This book is mainly devoted to anatomy and principles of medicine (temperaments, humors, general aspects of health), pharmacy, also, symptoms, signs, and treatment of diseases of the special organs is discussed in detail [12-14]. Furthermore, Tabari considered examination of pulse and urine and dental medicine, therefore “ *Firdous al- Hikmat fi al- Tibb ”* is a practical and technical book in medicine [13,14].

###  Clinical Description of “ Hayajan al-Dam”

In “ *Firdous al- Hikmat*,” Tabari explained that any direct or indirect change in the quantity/quality of each one of humors in the body’s systems could cause disease [14]. He described a condition due to change in “ *Dam*” humor and named it“ *Hayajan al-Dam*” (blood excitement), according to Tabari, this disorder can manifest itself with symptoms including the presence of red face, feeling hot in the body, *Imtila* (fullness) in the vessels, feeling sweetness in the mouth and oversleeping (a lot of sleep) [14]. *Imtila* in the vessels is considered as one of the symptoms of “ *Hayajan al-Dam*,” in this condition, quantity/quality of intravascular fluid changed beyond vessels’ capacity and increased total amount of blood volume [14-18]. The general symptoms of *Imtila* such as; heaviness in the head and headache, sluggish body movement, redness of the body, warmness of the body on touch, prominent, distended and tense veins, loss of appetite, blurred vision, yawing and drowsiness, difficulty concentration, dense and colored (turbid) urine, epistaxis, skin tension, Furthermore, increased vascular tension can lead to rupture and present with hemorrhage and sudden death [15-18]. According to historical medical manuscripts, clinical manifestations of“ *Hayajan al-Dam*” have also been defined later by other TPM scientists such as Rhazes [15], Akhawayni [6], Haly Abbas [17] and Avicenna [18], under the topic of *Imtila*, in the TPM textbooks, *Imtila* means accumulation of matter and humors in vessels and organs, it is divided into “ *imitial bi- hasabi -l- aw’iyah*” (quantitative change of blood composition, which causes the fullness of the canals and vessels of the body) and “ *imtila bi- hasabi -l- qowwah*” (qualitative change of blood composition) [15-18]. Comparison of clinical manifestations of “ *Hayajan al-Dam*” and HTN is shown in Table-1.

## Suggested Treatment

From the perspective of TPM, although there are common therapeutic plans for treatment of diseases, each method in each is exclusive [18]. Lifestyle modification, oral drugs, and phlebotomy or getting massage is suggested for treating this condition [14]. Tabari recommended the use of *Ziziphus jujube* ( *Annab*) can lead to alleviation of blood excitement [14], other TPM scholars pointed out the jujube can relieve the excitement of the blood [16-18]. Furthermore, Tabari recommended the *Annab* for treatment of the digestive disorder, headache, lung disease, and types of fevers [14].

## Discussion

In this article, we explained the concept of “ *Hayajan al-Dam*” according to Tabari’s opinion and evaluated it from the viewpoint of other TPM scholars, based on our findings; there are similarities between the “ *Hayajan al-Dam*” and “ *imtila bi- hasabi -l- aw’iyah*” [14-18]. It is clear that in TPM approach, pathophysiology and recommended treatments for any disease were based on humoral theories which cannot be easily compared by the current concept of medicine. Reviewing the main textbooks of TPM, indicate that there is no specific disease, which be considered exactly equivalent of HTN in the conventional medicine [14-18], although some similarities have existed between main clinical manifestations of “ *Hayajan al-Dam*” or *Imtila* and arterial HTN. In previous studies, clinical manifestations of arterial HTN compared with some diseases in main TPM textbooks to find a specific disease equivalent of HTN, and several hypotheses have been raised [6-10]. Emtiazy *et al. *have introduced Avicenna’s opinion about arterial HTN, the authors believed that Avicenna described most of the clinical manifestations and complications of HTN in the Canon of Medicine by details in *emtela bihasab -al- aw’eyyah* and *ghalabat -al- sauda* (Black bile dominance) chapters, they concluded that deposition of abnormal black bile in vascular wall reduces the elasticity of arteries and can cause high peripheral resistance [7]. Ghods *et al.*have pointed to Avicenna and Haly Abbas concepts in this context, their findings show that *Imila* ( *imtila bi- hasabi -l- aw’iyah*) has the most overlap with symptoms of HTN,they also proposed that other causes can lead to HTN including vascular wall dry dystemperament (atherosclerosis), cardiac hot dystemperament and damages of other body organs like liver, kidney and nervous system [6]. The concept of other TPM scholar named *Akhawayni Bokhari* on HTN was reviewed by Heidari *et al*.they concluded that the definition, clinical manifestations, and treatments of HTN presented under the topic of *Imtila* in the “ *Hidāyat al*- *Muta’allimin fi al*- *Tibb ”* (The Students’ Handbook of Medicine) [8]. Shamshad Ahmad considered that those symptoms have been described by Rhazes for *imtila bi- hasabi -l- aw’iyah* are similar to clinical features of HTN [9]. TPM scientists were familiar with other medical traditions like Unani medicine and had used their theories. Therefore, the history of HTN in other traditional medicine has also been reviewed [9,10]. Although some authors use the term “ *Zaghtuddam Qawi*” to describe HTN in Unani medicine, this term has not been considered in the classical Unani literature; it is believed that most of HTN manifestations were matched with *imtila bi- hasabi -l- aw’iyah* in those literature [9,10]. An accurate reviewing of main textbooks of TPM clarified that types of *imtila* like *imtila bi- hasabi -l - aw’iyah* is a general condition and did not considered as a distinct disease in special organ diseases, in fact, it is a condition can affect various parts of the human body then specific symptoms of the distinct disease be revealed, in those textbooks, *Imtila* is considered as a predisposing and precipitating factor for other specific diseases [15-18].

## Conclusion

It seems that arterial HTN is not equivalent of a specific disease in TPM; however, it may be considered as a complication of other diseases like cardiac and renal diseases, arterial diseases, and blood flow disorders. In previous studies, several hypotheses have been made to achieve the etiology of HTN in TPM sources, most of them presume that clinical features of HTN are as the same as *imtila bi- hasabi -l- aw’iyah, *also other diseases are also proposed such as black bile dominance, vascular wall dry dystemperament, cardiac hot dystemperament and damages of other body organs like liver, kidney, and nervous system, finally, based on the basics of TPM, we cannot confirm these results, we strongly recommend doing more clinical and experimental studies to achieve better clinical outcomes.

## Acknowledgment

 The authors wish to thank, School of Medicine, Zanjan University of Medical Sciences.

## Conflict of interest

 No conflict.

**Table 1:** Comparing the Symptoms of *Hayajan al-Dam* and HTN

***Hayajan*** *** al-*** ***Dam*** [14]	**Hypertension** [19]
• Headache with heaviness	• Morning headache
• Heaviness of body, sluggish body movements	• Early fatigue and muscle weakness
• Blurred vision	• Blurred vision
• Turbid urine	• Hematuria
• Distended vessels, fullness of the vessels and possibility of rupture	• Aortic dissection, leaks of aneurysms
• Sanguinary stroke	• Transient ischemic or hemorrhagic stroke
• Nasal bleeding	• Epistaxis
• Oversleeping	• Insomnia
• Loss of appetite	• Anorexia
• Presence of red face, feeling hot in the body	• Flushing (feverish), which is characteristic of pheochromocytoma and severe HTN
• Feeling skin tension	• Nausea
• Feeling sweetness in the mouth	• Sexual incompetency
